# 

**Published:** 1907-06

**Authors:** 


Sixty-second Year.

VOL. LXII. JUNE, 1907. NO. 11

### BUFFALO MEDICAL JOURNAL

Established 1845 by AUSTIN FLINT, M. D.

### NOTICE OF REMOVAL

**The Editorial Offices of this Journal have been removed to Delaware Court, 238 Delaware Avenue, where all communications, correspondence, exchanges and books for review should be addressed**

***William Warren Potter.***

